# Draft genome sequence of *Staphylococcus capitis* strain 13-29 isolated from the human nasal cavity

**DOI:** 10.1128/mra.00141-26

**Published:** 2026-04-20

**Authors:** Ken-ichi Okuda

**Affiliations:** 1 Department of Life, Environment and Applied Chemistry, Fukuoka Institute of Technology 53335, Fukuoka, Japan; University of Pittsburgh School of Medicine 12317 , Pittsburgh, Pennsylvania, USA

**Keywords:** *Staphylococcus capitis*, draft genome, coagulase-negative staphylococci

## Abstract

The draft genome sequence of *Staphylococcus capitis* strain 13-29, isolated from the human nasal cavity in 2017, is reported. The genome was sequenced using the PacBio RS II platform and assembled into 5 contigs totaling 2,544,103 bp with a GC content of 32.9%.

## ANNOUNCEMENT

*Staphylococcus capitis* is a gram-positive, coagulase-negative staphylococcus that is generally regarded as a commensal organism but is also an opportunistic pathogen, particularly in clinical settings (1, 2). *S. capitis* is an important cause of neonatal sepsis and other healthcare-associated infections, and genomic diversity has been reported among clinical isolates (3, 4). Genome sequence information is essential for understanding ecological adaptation, antimicrobial resistance, and pathogenicity in this species.

The draft genome sequence of *S. capitis* strain 13-29, isolated from a human nasal swab in 2017, was determined. The swab specimen was streaked onto brain heart infusion (BHI) agar and incubated aerobically at 37°C for 24 h. Genomic DNA was extracted from cells grown overnight in BHI broth using the Wizard Genomic DNA Purification Kit (Promega) according to the manufacturer’s instructions.

Long-read sequencing was performed using the PacBio RS II platform. Genomic DNA was sheared using g-TUBE (Covaris), and a 20 kb SMRTbell library was prepared using the SMRTbell Template Prep Kit 1.0 (Pacific Biosciences). DNA fragments were size-selected using the BluePippin system (Sage Science) with a 15 kb cutoff to enrich fragments >15 kb. Sequencing was performed using one SMRT Cell with P6-C4 chemistry. In total, 111,251 polymerase reads and 142,051 subreads (1,701,020,040 bp) were generated; the subread length N50 was 16,771 bp, providing approximately 670-fold genome coverage based on the final assembly size. Reads were processed in SMRT Analysis version 2.3.0 to remove SMRTbell adapters and generate filtered subreads.

*De novo* genome assembly was performed using the HGAP Assembly.3 pipeline implemented in SMRT Analysis version 2.3.0 (5). HGAP Assembly.3 includes preassembly error correction of long reads prior to contig construction. The assembled contigs were further processed using Circlator version 1.2.1 (6). Contigs fully contained within other contigs were removed. Contigs with overlapping ends were circularized, and the genome start position was rotated using the dnaA gene sequence. Consensus polishing was performed with Quiver (RS_Resequencing.1) in SMRT Analysis (5). During polishing, PacBio subreads were mapped to the curated contigs, and the consensus sequences were corrected based on the mapping results. Default parameters were used except where otherwise noted. Species identity was confirmed by BLASTn analysis (NCBI) of the 16S rRNA gene sequence (1,548 bp) extracted from the genome assembly; the best hit was *Staphylococcus capitis* strain BN2 (GenBank accession no. CP042341.1) with 100% identity (1,548/1,548) and 0 gaps.

Key genome features of *Staphylococcus capitis* strain 13-29 are summarized in Table 1. The resulting draft genome assembly consisted of 5 contigs with a total length of 2,544,103 bp and an overall GC content of 32.9%. The largest contig length and the assembly N50 were both 2,462,918 bp, indicating a high-quality draft genome assembly with high contiguity. Genome annotation was performed using the DFAST annotation pipeline (7), which predicted 2,456 protein-coding sequences, 19 rRNA genes, and 62 tRNA genes. The availability of this genome sequence will facilitate comparative genomic analyses of *S. capitis* and other coagulase-negative staphylococci and will support future investigations into the evolution and host adaptation of this species (8–10).

**TABLE 1:** Genome features of *Staphylococcus capitis* strain 13-29

Feature	Value
Total length (bp)	2,544,103
Number of contigs	5
GC content (%)	32.9
Assembly N50 (bp)	2,462,918
Protein-coding sequences	2,456
rRNA genes	19
tRNA genes	62
CRISPR arrays	0
Coding ratio (%)	85.2

## Data Availability

The draft genome sequence of *Staphylococcus capitis* strain 13-29 has been deposited in DDBJ/ENA/GenBank under the accession numbers BAAJXU010000001, BAAJXU010000002, BAAJXU010000003, BAAJXU010000004, and BAAJXU010000005. The BioProject and BioSample accession numbers are PRJDB40238 and SAMD01814116, respectively. The raw PacBio sequencing reads have been deposited in the DDBJ Sequence Read Archive (DRA) under the accession number DRR902766. The DDBJ Sequence Read Archive is a public repository providing integrated access to raw sequencing data within the International Nucleotide Sequence Database Collaboration (INSDC) (11).
